# Accurate Mobile Urban Mapping via Digital Map-Based SLAM †

**DOI:** 10.3390/s16081315

**Published:** 2016-08-18

**Authors:** Hyunchul Roh, Jinyong Jeong, Younggun Cho, Ayoung Kim

**Affiliations:** 1 Robotics Program, Korea Advanced Institute of Science and Technology, Daejeon 34141, Korea; rohs_@kaist.ac.kr; 2 Department of Civil and Environmental Engineering, Korea Advanced Institute of Science and Technology, Daejeon 34141, Korea; jjy0923@kaist.ac.kr (J.J.), yg.cho@kaist.ac.kr (Y.C.)

**Keywords:** 3D mapping, SLAM, digital map, urban mapping system, IPM, lane map

## Abstract

This paper presents accurate urban map generation using digital map-based Simultaneous Localization and Mapping (SLAM). Throughout this work, our main objective is generating a 3D and lane map aiming for sub-meter accuracy. In conventional mapping approaches, achieving extremely high accuracy was performed by either (*i*) exploiting costly airborne sensors or (*ii*) surveying with a static mapping system in a stationary platform. Mobile scanning systems recently have gathered popularity but are mostly limited by the availability of the Global Positioning System (GPS). We focus on the fact that the availability of GPS and urban structures are both sporadic but complementary. By modeling both GPS and digital map data as measurements and integrating them with other sensor measurements, we leverage SLAM for an accurate mobile mapping system. Our proposed algorithm generates an efficient graph SLAM and achieves a framework running in real-time and targeting sub-meter accuracy with a mobile platform. Integrated with the SLAM framework, we implement a motion-adaptive model for the Inverse Perspective Mapping (IPM). Using motion estimation derived from SLAM, the experimental results show that the proposed approaches provide stable bird’s-eye view images, even with significant motion during the drive. Our real-time map generation framework is validated via a long-distance urban test and evaluated at randomly sampled points using Real-Time Kinematic (RTK)-GPS.

## 1. Introduction

The recent development of autonomous vehicles encompasses many key issues in robotics problems, including perception, planning, control, localization and mapping. Among these problems, this paper focuses on solutions for an accurate urban map generation, specifically targeting 3D and lane maps for autonomous cars. Having an accurate map is an important issue for self-driving cars [1,2,3] and many other areas such as virtual reality [4], visualization [5], recognition [6], localization and navigation [7,8].

In conventional mapping approaches, aerial sensing has been heavily investigated. In these approaches, the fusion of aerial images with aerial Light Detection and Ranging (LiDAR) and/or radar is usually applied for an accurate digital map at the cm-level, including urban structures. This line of studies focuses on undistorting building shapes from aerial sensors, which is mostly too costly (ranging from $0.5 M to $1.4 M). To obtain cost-effective solutions for mapping, recent advances have appeared in mobile mapping systems. Using a car-like system with sensors mounted, 3D maps are often generated from a set of point clouds, using LiDAR sensors to actively acquire accurate and dense 3D point clouds of building façades or surfaces of objects. Mobile scanning is widely used for modeling in architecture [4] and agriculture [9], as well as for urban and regional planning [10]. Many studies combine a highly accurate GPS, a high precision Inertial Measurement Unit (IMU) and the wheel/visual odometry of the vehicle to compute fully timestamped trajectories. The absence of the GPS in constricted spaces is an exceptionally limiting factor for mobile scanning. It is more critical, in a complex urban environment, and thus it is difficult to calculate accurate positions using a GPS sensor due to blackouts or multipath problems. Generally, using a variety of sensors has became a popular strategy to complement the drawbacks of a single sensor.

We propose a SLAM-based approach to localize the mobile sensor system mounted on a car-like platform and generate an accurate urban map consisting of a large amount of 3D points. For accurate localization, we use publicly available digital maps together with cameras, Light Detection and Ranging (LiDAR)s, and inertial sensors. Using this accurate localization, motion compensated Inverse Perspective Mapping (IPM) is applied for accurate lane-map generation. Given accurate localization, projections of front-looking cameras follow the construction of the lane map. For the lane map description, we propose an adaptive IPM algorithm to obtain accurate bird’s-eye view images from the sequential images of forward-looking cameras. These images are often distorted by the motion of the vehicle; even a small motion can cause a substantial effect on bird’s-eye view images.

To use a digital map in Simultaneous Localization and Mapping (SLAM), we incorporate a shape file to extract structural and elevation information. Although digital maps with 1:1000 and 1:5000 scales offer sub-meter global average accuracy, only a subset of map data (e.g., buildings and road boundaries which are globally static) are available for vehicle localization. In this paper, we introduce a measurement model for both GPS and structural information, and leverage the digital map data as observations to actively fuse digital maps in SLAM when GPS signals are sporadic.

The contribution of the proposed method can be summarized as follows.

- Digital map-based SLAM: Digital map information was incorporated with a building model, road network and elevation model. This paper introduces a measurement model to add global measurements for full 3D SLAM. Instead of addressing this problem as localization to a prior map, our modeling handles the structural information in the digital map as sporadic spatial measurements.
- Development of online mobile mapping system with sub-meter accuracy: Typical LiDAR-based mapping system known to be slow, suffering from association problems. Despite a large number of nodes and a long trajectory, the entire method runs in real-time using only 40% of the total mission time. Not only the computational time, our algorithm provides memory-efficient implementation. Wall information obtained from point cloud turns into an urban signature, which then can effectively be used in the matching phase. Fully integrated single step implementation allows us to build a 3D map over 9 km while driving.
- Thorough analysis for resulting 3D and lane maps accuracy: We perform a thorough analysis on the resulting maps to evaluate the effects of digital maps. The addition of elevation and buildings from digital maps enables accurate 3D urban mapping. We performed quantitative evaluation on random sample points. Using RTK-GPS with 10-mm-accuracy, we analyzed the accuracy of the points from buildings and lane maps.

## 2. Related Works

Conventional approaches in digital map generation rely on aerial sensors, including high-resolution aerial images, Light Detection and Ranging (LiDAR)s, and radars. Toward accurate building detection, point cloud data from aerial LiDAR has been widely used for a Digital Building Model (DBM) [11,12,13,14]. One line of study used only aerial imagery to extract building information [15,16], while merging the high-resolution aerial imagery and aerial LiDAR [17] has been introduced.

The aforementioned approaches use costly aerial sensors and have limitations in generating maps inside tunnels and multi-layered roads. For a cost-effective mobile mapping system, urban mapping systems similar to ours have been introduced in the literature [18,19,20,21]. Blanco et al. [18] presented a collection of outdoor datasets using a large and heterogeneous set of sensors comprising color cameras, several laser scanners, precise GPSs, and an IMU. Their posterior research [19] introduced a dataset gathered entirely in urban scenarios using a car equipped with one stereo camera and five laser scanners, among other sensors. Elseberg et al. [20] used a commercial data logging system and collected data with an experimental platform constructed by the RIEGL scanner [22]. Bok et al. [21] used a mobile mapping sensor system mounted on a ground vehicle. A vertical 2D LiDAR scans structures and the reconstruction is done by accumulating scanned data. All of the conventional approaches cover a small region, assuming a 2D environment without taking altitude information into account.

For these mobile mapping systems, cameras are the most popular sensors in detecting lanes. For visual road-mark detection, IPM is often used in the vision-based perception of roads and lanes. IPM produces bird’s eye-view images that remove the perspective effect by using information about camera parameters and the relationship between the camera and the ground. The results of IPM can provide post-processing algorithms with more efficient information, such as lane perception, mapping, localization, and pattern recognition. Many researchers have studied IPM in many applications, such as distance detection [23], production of bird’s eye-view images of a spacious area using a mosaic method [24], provision of appropriate bird’s eye-view images for parking assistance [25], and lane-level map generation [26].

The challenge for mobile mapping systems is to achieve a consistent map. Researchers investigated the SLAM algorithm for accurate vehicle localization [27] and 3D mapping [28] using perceptual sensors mounted on a vehicle. Using a digital map has also been recently investigated by many researchers [29,30,31]. Schindler et al. [29] first proposed lane map generation and representation methods using RTK GPS-based localization and showed high-precision digital map-based self-localization in [30]. They showed an efficient Monte Carlo localization approach using a map model based on smooth arc splines. Floros et al. [31] proposed an approach for global vehicle pose estimation that combines visual odometry with map information from OpenStreetMaps [32] to provide accurate estimates of the vehicle’s pose. Similarly, Pink and Stiller [33] described landmark (lane-based) generation from aerial images with image classification geometric representation. This method used aerial images for prior maps and focused on the way to match orthographic lane images to a global lane map from aerial images. Guo et al. [26] presented a system for lane-level map generation from local IPM images and the global OpenStreetMap (OSM) database. They locally constructed an orthographic lane map, matched it with a segmented aerial map, and finally generated a global lane graph of real-world roads.

As an accurate localization aspect, some previous works [34,35,36] used a lane map as a local vehicle position estimation by matching pre-built local lane maps from IPM images and current incoming raw images. Napier and Newman [34] proposed a vehicle localization method based on synthesized local orthographic lane maps. They generated local orthographic images from a first run and localized the vehicle by Mutual Information (MI) between live-stream images and synthesized images. Schreiber et al. [35] constructed prior lane-based maps with extended sensors such as GPS/INS and matched road markings and curbs for lane-level localization. Rose et al. [36] used lane maps for lateral position estimations of a vehicle. These studies only applied localization or pose estimation algorithms; no cases have generated 3D maps using the SLAM algorithm with a digital map. In this paper, we are interested in using publicly available digital maps in a SLAM framework to leverage for accurate lane map and 3D map generation.

A similar approach to ours was reported in [37] which incorporated bundle adjustment using monocular vision. Although extracting buildings from digital maps and using them as constraints were introduced and conceptually similar to ours, their implementation relied mostly on vision, covering a relatively small urban area. Unlike their approach, proposed algorithm focuses on using LiDAR accomplishing real-time performance with a single-step mapping algorithm that can build an urban map while driving.

## 3. System Overview

**Figure 1:**
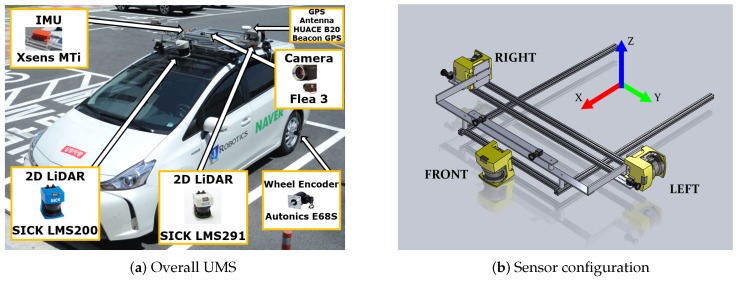
Sensor system description. Our sensor suite includes two 2D LiDARs, four cameras, a GPS, two wheel encoders, an IMU, and an altimeter. Note that the front LiDAR in (**b**) is only for moving object detection, and thus excluded in Simultaneous Localization and Mapping (SLAM) framework.

For accurate urban mapping, we developed a sensor suite called Urban Mapping System (UMS), which is mountable to a car-like platform. As shown in Figure 1a, the platform used for mapping was equipped with three Light Detection and Ranging (LiDAR) sensors, four cameras, a Global Positioning System (GPS), an Inertial Measurement Unit (IMU), an altimeter, and wheel encoders. The detailed specifications are summarized in Table 1. As in Figure 1, coordinates of each sensor were represented with respect to the vehicle center coordinates (red, green and blue arrows).

Using a geometrical relationship between the vehicle center and LiDAR, the position of the camera is computed using extrinsic calibration results of LiDAR and cameras. For more details, refer to [38] the system configuration.

In the proposed configuration, LiDAR sensors are facing each side of the vehicle, while the sweeping direction is orthogonal to the ground. This configuration allows us to capture surrounding data line-by-line. Motion induces accumulation of lines, and a 3D point cloud is obtained by stacking a sequence of point cloud lines. Researchers using similar configurations recommended a vehicle speed of approximately 15–30 km/h [39], which we also adopted in the experiments.

**Figure 2:**
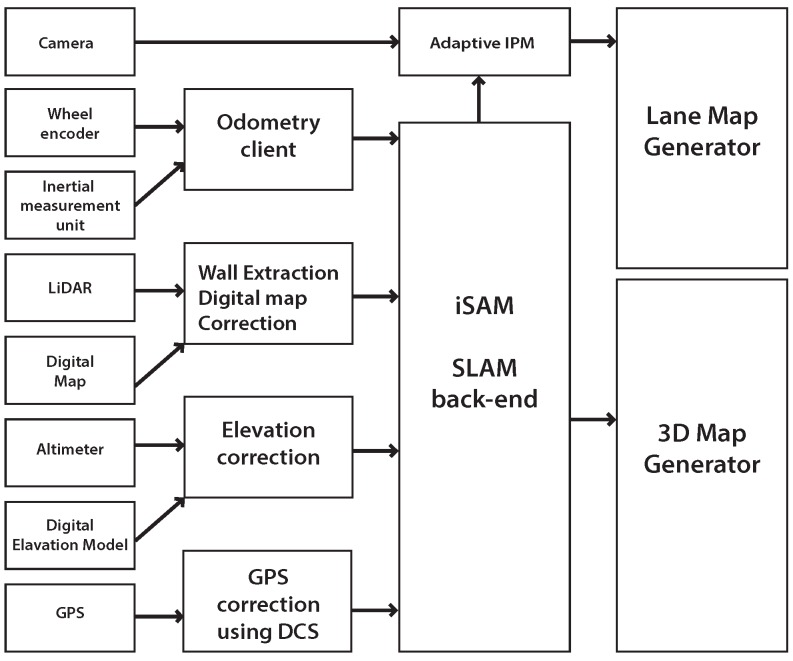
Software design diagram. The 3D point cloud map generation involves four main steps.

The objective of the proposed platform is to build an accurate 3D and lane map given various sensors and digital maps. The process of generating urban lane maps with 3D point clouds involves four main stepsas shown in Figure 2: (1) sensor data logging; (2) generating a node and the factor; (3) optimizing using an Incremental Smoothing and Mapping (iSAM) SLAM back-end; and (4) generating the final lane map based on the optimized trajectory from the SLAM results and IPM images. Measurements from odometry, digital map information, elevation, and GPS are all used.

## 4. Pose-Graph Simultaneous Localization and Mapping (SLAM)

### 4.1. State Definition

**Figure 3:**
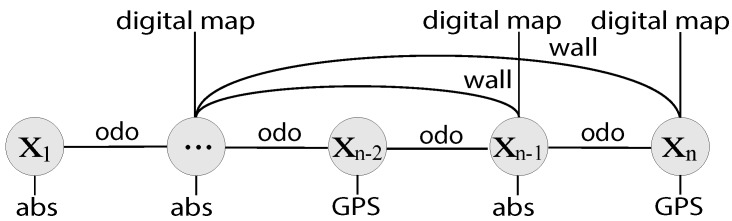
Depiction of the pose-graph SLAM factors. Odometry constraints (odo) are sequential whereas wall loop constraints (wall) can be non-sequential. Absolute constraints (abs) includes digital map correction and altimeter. GPS factors may occur irregularly. When wall is created, we also provide a digital map correction factor.

We estimate the vehicle’s full 6-degree of freedom (DOF) pose, $x={\left[x,y,z,\phi ,\theta ,\psi \right]}^{⊤}$. The augmented state representation is expressed as follows for *n* keyframes,

(1) $$X={\left[\begin{array}{c} x_{1}^{⊤},\cdots ,x_{i}^{⊤},\cdots ,x_{n}^{⊤} \end{array}\right]}^{⊤}$$

while each of the pose samples ($x_{i}$) correspond to the time instance $t_{i}$ of a keyframe. For the SLAM back-end, we use iSAM [40] to find an optimized solution of the trajectory, from which we build the urban map. Factors from each sensor measurement are collected as in Figure 3. The attitude measurements and altitude are given as absolute factors per node, with all nodes sequentially connected by odometry measurements. Loop-closing factors are mainly generated by walls that we generate from LiDAR when we add building measurement from digital maps. GPS availability is quite sporadic in urban area, and measurements are intermittently applied to a node using Dynamic Covariance Scaling (DCS).

### 4.2. Odometry Modeling

The system has two rotary encoders, as shown in Figure 1a, which are mounted to record wheel revolution counts and the resulting rotation angles ($C_{l}$ and $C_{r}$) of each wheel. We define vehicle pose in 2D space, $x={\left[x,y,\theta \right]}^{⊤}$ for odometry measurements. We calculate the relative pose difference as a function of left/right wheel diameter ($D_{l},D_{r}$), left and right wheel rotation angle ($C_{l}$ and $C_{r}$), wheel diameter ($D_{l},D_{r}$), and wheel base ($W_{b}$).

(2) $$\begin{array}{ccc} z_{odo} & = & \left[\begin{array}{c} \Delta x\\ \Delta y\\ \Delta \theta \end{array}\right]=\left[\begin{array}{c} L_{avg}\cos\left(\Delta \theta \right)\\ L_{avg}\sin\left(\Delta \theta \right)\\ L_{diff}W_{b}^{-1} \end{array}\right] \end{array}$$

Here, $L_{avg}$ is the average and $L_{diff}$ is the difference of two travel distances from the left and right wheel.

### 4.3. Altitude and IMU Modeling

We also provide z-directional measurements from the altitude sensor. When mapping hilly terrain, this leverages the localization performance by providing both absolute and relative measurements on height. We used theWITHROBOT MyPressure (WITHROBOT, Seoul, Korea) altitude sensor [41], which has accuracy within 1 cm. Attitude information was obtained via IMU. A MTI Xsens [42] was mounted into the system to provide roll, pitch, and yaw in 100 Hz. Altitude and attitude information is used as absolute measurement factors and fed into the SLAM back-end.

The proposed implementation produces loop-closure on height term based on wall information Section 5.3. When a loop-closure happens in wall-to-wall matching (i.e., when a wall is revisited), we apply a relative constraint on two associated nodes so as for them to be the same height.

### 4.4. GPS Modeling

GPS becomes extremely unreliable in highly complex urban environments. Signals are lost or deteriorate in urban canyons. Despite these limitations, GPS still provides valuable information to ground vehicles. For our GPS measurement error modeling, we mainly used the GPS Pseudorange Noise Statistics (GPGST), which include the standard deviation of longitude/latitude errors. The error measurement is the most sensitive, depending on the complexity of the environment, showing high error values in complex urban area.

Outlier handling in SLAM determines the robustness of the entire framework, since a single wrong measurement may critically deteriorate the results. To tackle this issue, Sünderhauf and Protzel [43] introduced Switchable Constraints (SC) and switching variables ($S_{ij}\in \left[0,1\right]$) for the robust SLAM back-end. This research area was further developed by Agarwal et al. [44], who solve for a closed form solution to determine these switching variables $S_{ij}=\min.\left(1,\frac{2\Phi }{\Phi +\chi_{ij}^{2}}\right)$ As can be seen, the choice depends on $\chi_{ij}^{2}$, which represents the error for each loop closing constraint. By considering the induced error by a factor, the associated covariance is dynamically scaled. This substantially improves the results when a certain measurement may be unreliable during the mission, such as GPS. In this implementation, we adopted DCS to cope with intermittent and unreliable GPS signals.

## 5. Digital Map-Based SLAM

We introduce using a digital map with Light Detection and Ranging (LiDAR) sensor to correct the navigation error even under unreliable Global Positioning System (GPS). We first create walls from a 3D point cloud generated by LiDAR. Using this point cloud as a prior reference, the building edges are automatically detected to correct navigation error. In this section, we introduce the generation of walls, matching with a digital map, and matching between walls for loop-closure.

### 5.1. Digital Map

We leverage publicly available digital maps for SLAM application. Specifically, we focus on vectorized digital maps widely used in a geospatial information system. Studies that automatically generate the digital map have been continued in recent years [11,12,13,14,15,16,17,45,46,47,48] but it has mostly relied on aerial sensors. For the digital map data format, we chose to use a shapefile format developed by ESRI [49]. The shapefile stores non-topological geometry and attribute information for the spatial features as a set of vector coordinates. Vector features including points, lines, and polygons are described in the shapefile format. The format also represents area features via closed loop and double-digitized polygons. Each attribute record has a one-to-one relationship with the associated shape record. Among many types of records, we focus on polygons that contain information about structural buildings.

**Figure 4:**
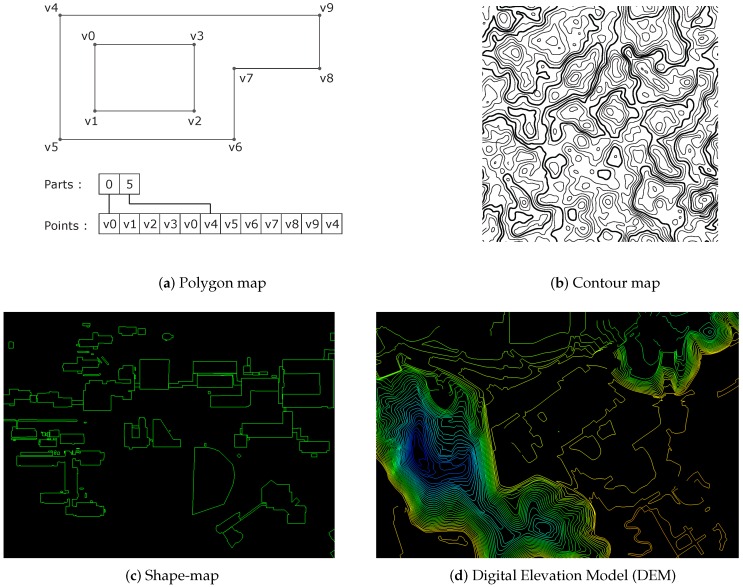
Shape file representations for building and contour. An example of polygon data type structure is in (**a**), and an example of contour map is in (**b**). (**c**) is sample shape-map (building) in the campus data. (**d**) is sample shape-map (DEM) in the campus data.

**Figure 5:**
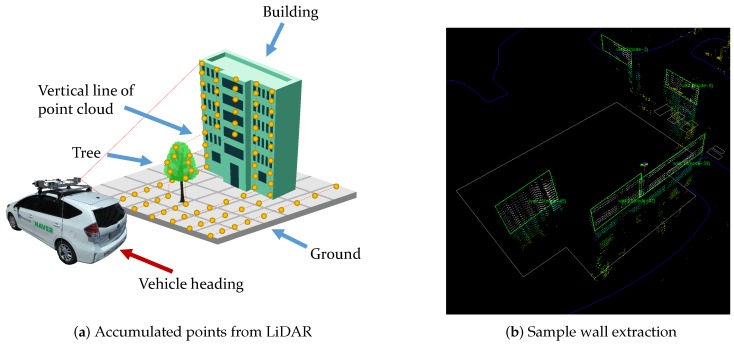
(**a**) Illustration of point cloud accumulation from vertically installed side scanning LiDAR. We only collect a single scan line per vehicle pose, and accumulate lines into a local point cloud set via motion. (**b**) An example of wall represented by digital map and point cloud map (white points in green box). For wall segmentation, we use 5-point Random Sample Consensus (RANSAC)-based plane fitting.

As illustrated in Figure 4a, a polygon consists of one or points that form a closed, non-self-intersecting loop. A polygon may contain more closed curves. A closed curve is a connected sequence of four or more multiple outer closed curves. The order of vertices or orientation for a closed curve indicates which side of the closed curve is the interior of the polygon. Recording contents of this polygon data type are shown in Table 2. Figure 5b presents an example of a building extracted from polygon data in a shapefile. In this paper, shapefiles from National Geographic Informaion Institue (NGII) are used for building extraction.

We also incorporate DEM for elevation information. For certain topographies, regions contain substantial elevation change. In general, the height of the terrain is represented by the contours. As presented in Figure 4d, points are connected by contour lines indicating the same height. The absolute height value of the contour is described in the attribute information of the shape-file. When the trajectory of the vehicle passes a contour, SLAM algorithm optimize position using absolute measurement of Z-direction from this absolute height.

### 5.2. Wall Segmentation

Given building information parsed from the digital maps, we now extract walls from the mobile sensor system. Building façades and other structures’ surfaces are major features of the urban area. As digital maps are widely available these days, we propose a matching algorithm that uses a digital map and an accumulated local point cloud. In general, use of the point-to-point Iterated Closest Point (ICP) algorithm is popular but it is time-consuming and gives rise to issues when point clouds are not sufficiently dense.

We define a point cloud $P=\{p_{1},p_{2},p_{3},...\}$ as consisting of LiDAR points, while each point indicates a 3D position $p_{k}=\left[x_{k},y_{k},z_{k}\right]$ in the global coordinate frame. From the LiDAR 3D point cloud in the sensor frame, ($P_{s}$) is obtained by converting each beam ranging from i=0 to 360. The angular resolution of SICK LMS291 is $N_{res}=0.5^{\circ }$, covering a $180^{\circ }$ field of view for each size. Sensor frame point cloud $P_{s}$ is then converted to vehicle frame point cloud $P_{v}$ using a coordinate transformation.

After the transformation, we obtain a vertical line of the point cloud as described in Figure 5a. As the vehicle proceeds, these lines are accumulated and form a local point cloud set. Given a single scan line of LiDAR and odometry information from the wheel encoder and IMU, the set of the line forms a local point cloud-based on the estimated pose. During the local point cloud forming, we evaluate the wall criteria to see if the set can be segmented to a wall. When a wall is registered, the algorithm creates a node in the SLAM graph as a keyframe. Dropping the node clears the accumulated local point cloud and starts a new accumulation of incoming scan lines.

**Figure 6:**
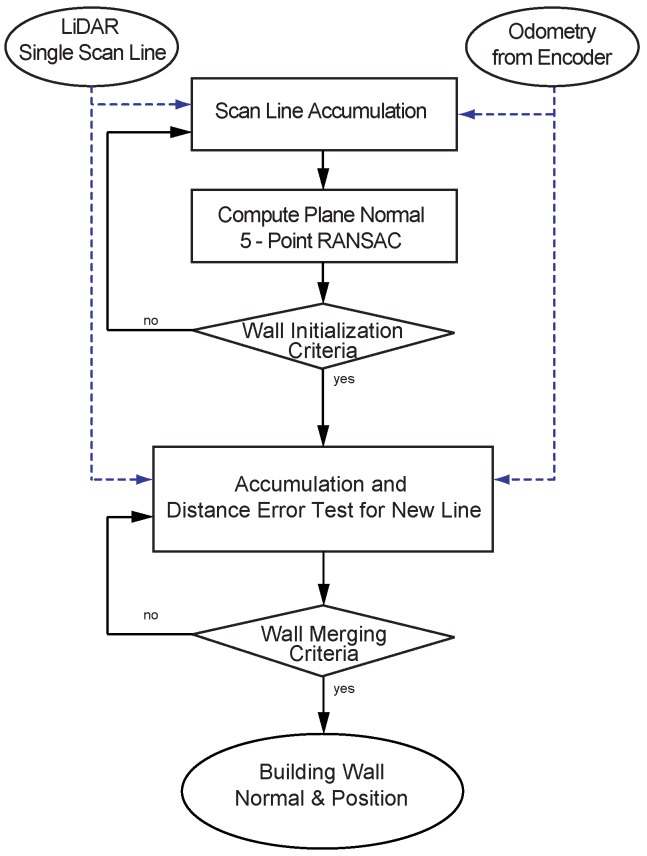
Block diagram for wall segmentation. The overall wall extraction algorithm can be divided into two parts. The first part is to obtain the initial normal, the second is a part of the segmentation wall to error distance test of the new incoming scan line by using initial normal information.

We propose a fast wall segmentation algorithm-based on RANSAC when segmenting a wall from a point cloud (summarized in Figure 6). For the incoming line of the point cloud, we fit a plane using a 5-point RANSAC. Our wall initialization criteria involve checking the wall width (initialization criteria in Figure 6). If the width of the created wall reaches our threshold (minimum 3 m), the algorithm initializes a wall and stores normal vector and error statistics for the wall. Once the initial wall is established, a newly incoming scan line is evaluated to be merged to the initial wall (merging criteria in Figure 6).

### 5.3. Wall-to-wall Loop Closing

For loop-closure, we use building wall patches by introducing a wall-to-wall measurement model. This method provides an explicit relationship between the walls in the factor graph and produces efficient maps using a data-rich 3D point cloud.

We parameterize the wall ${ß}_{i}={\left[n_{i}^{x},n_{i}^{y},n_{i}^{z},p_{i}^{x},p_{i}^{y},p_{i}^{z}\right]}^{⊤}$ using normal vector $n_{i}={\left[n_{i}^{x},n_{i}^{y},n_{i}^{z}\right]}^{⊤}$ and the center point of the wall $p_{i}={\left[p_{i}^{x},p_{i}^{y},p_{i}^{z}\right]}^{⊤}$. Following wall coordinate transform operators ⊞ and ⊟ introduced by Paul Ozog [50] we derive our wall-to-wall measurement model. The ⊞ and ⊟ operations change the base coordinate frame that a plane is described in, which can be written as ${ß}_{jk}=x_{ij}⊟{ß}_{ik}$ and $x_{ij}⊞{ß}_{jk}=⊖x_{ij}⊟{ß}_{jk}$.

For two walls to be recognized as the same wall, they need to have the same normal vectors and the distance between a point in one plane to another plane should be zero. We compute them by evaluating the dot-product of two normals ($e_{n}$) and the distance from a center point on one wall to another wall ($e_{d}$).

(3) $$z_{wall-wall}={\left[\begin{array}{c} e_{n},e_{d} \end{array}\right]}^{⊤}$$

Let a wall *k* seen at frame *i* to be ${ß}_{ik}$ and the same wall revisited at frame *j* to be ${ß}_{jk}$. To compare normal vectors, ${ß}_{ik}$ needs to be transformed to frame *j* using ⊟ operation. For simple notation we denote this as ${ß}_{jk}^{\prime }=x_{ij}⊟\pi_{ik}$ with associated normal $n_{jk}^{\prime }$ and the center point $p_{jk}^{\prime }$. Then the error between two normal vectors can be written as $e_{n}=1-{\left(n_{jk}^{\prime }\right)}^{⊤}n_{jk}$. Similarly, the plane to point distance ($e_{d}$) is computed by measuring distance from ${ß}_{jk}^{\prime }$ to a wall center point in *j* frame ($p_{jk}$).

It should be noted that we intentionally excluded the center point’s coincident constraints. This is because our point cloud density strongly depends on the vehicle’s motion as we accumulate line scans. When a vehicle takes turns the density may increase or decrease, and the computed center point may be varying with respect to the accumulated point cloud density.

### 5.4. Wall-Based Digital Map Localization

With odometry having a moderate accuracy, structural information from buildings can be used as a feature to correct the accumulated navigation error. However, buildings usually deteriorate the GPS signal reception. For a complex urban area, therefore, GPS and buildings can be exploited complementary. The digital map contains a variety of local information and there are vectorized building data among them.

An extracted wall from a point cloud represented by ${ß}_{i}$ consists of normal vector $n_{i}\;=\;{\left[n_{i}^{x},n_{i}^{y},n_{i}^{z}\right]}^{⊤}$ and the center point of the wall $p_{i}={\left[p_{i}^{x},p_{i}^{y},p_{i}^{z}\right]}^{⊤}$.

Similarly, we can calculate wall information that consists of normal vector and a center position from digital map $d_{i}$ in global coordinates. Using ⊞ operation and current node pose, we write extracted wall information from point cloud as ${ß}_{ik}=x_{ij}⊞{ß}_{jk}$.

We obtain the measurement which consists of the dot-product of two normals ($e_{n}$) and the distance from a center point on one wall to another wall ($e_{d}$) in the same manner as in the Section 5.3.

(4) $$z_{wall-digitalmap}={\left[\begin{array}{c} e_{n},e_{d} \end{array}\right]}^{⊤}$$

### 5.5. Elevation-based Full 3D Mapping

For full 3D mapping, we use DEM that consists of contour lines by introducing a node-to-contour measurement model. When the vehicle traverses a contour, the algorithm detects an intersection between vehicle motion vector and a line segment from the contour.

**Figure 7:**
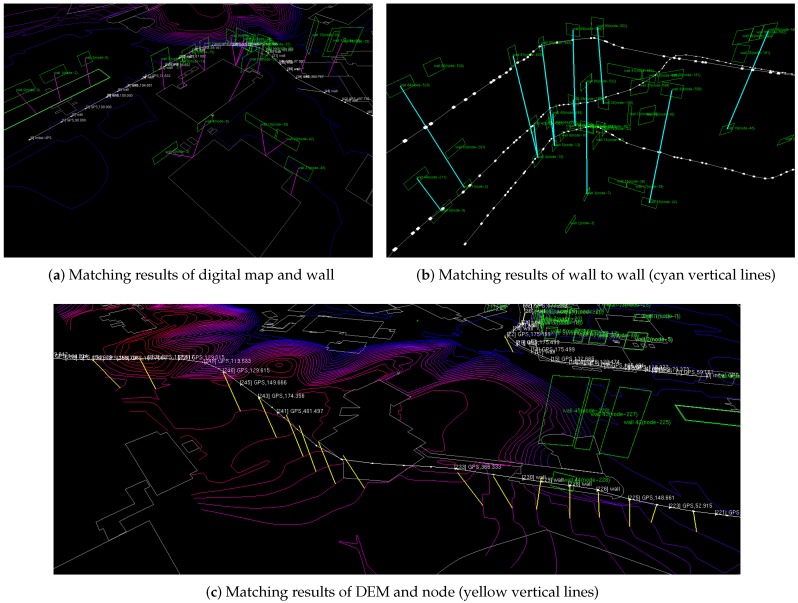

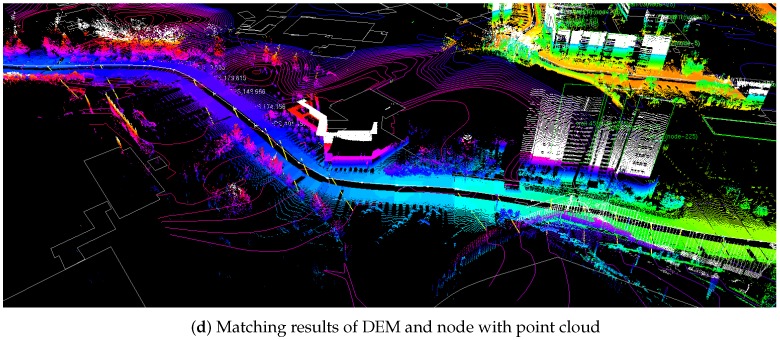
Result of digital map-based SLAM (**a**) Magenta line represents the correspondence of the digital map and building wall (green) extracted by point cloud. (**b**) Loop closure of trajectory generated using wall to wall matching showed by cyan line. (**c**) Correcting in the Z-axis direction, we use matching of Digital Elevation Model (DEM) and SLAM node. the yellow line represents this correspondence. (**d**) We can confirm the z-directional trajectory over the change of color.

Let the vehicle pose be $x={\left[x,y,z,\phi ,\theta ,\psi \right]}^{⊤}$. The DEM contour line, c, consists of vertices $c_{i}={\left[c_{i}^{x},c_{i}^{y},c_{i}^{z}\right]}^{⊤}$. Then we can extract two line segments, vehicle motion induced line ($l_{x}=\left[x_{i-1},y_{i-1},z_{i-1},x_{i},y_{i},z_{i}\right]$) that connects vehicle current node and previous node and contour line segment ($l_{c}=\left[c_{k-1}^{x},c_{k-1}^{y},c_{k-1}^{z},c_{k}^{x},c_{k}^{y},c_{k}^{z}\right]$) from a nearby contour. Using these two segments, we calculate the intersection point of these two line segments to determine the case when vehicle traverses contour line. Given vehicle pose that meets the DEM, our proposed method produces absolute measurement, which is described in the attribute information of the shape-file (Figure 7c).

## 6. Motion-Compensated Adaptive Lane Map Generation

**Figure 8:**
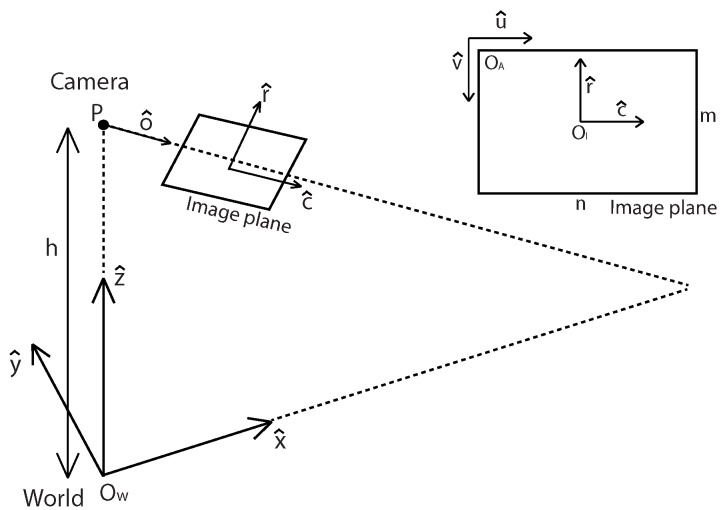
Coordinate the relationship between the camera, image and world. The unit of ($\hat{u}$, $\hat{v}$) is the pixel measurement, and that of ($\hat{r}$, $\hat{c}$) and ($\hat{X},\hat{Y},\hat{Z}$) is the meter measurement. Reprinted, with permission, from Jeong et al. URAI 2016; ©2016 IEEE.

Once we achieved an accurate localization by using a digital map-based SLAM, we apply IPM to back project road images onto the SLAM-induced map. This section briefly presents the basic IPM model [51] by using the physical parameters of a camera before illustrating an adaptive IPM model. IPM is a mathematical technique that relates to coordinate systems with different perspectives. More detailed derivation and explanation can be found in [52]. For lane map generation, we relate undisturbed bird’s-eye view images and forward-facing distorted images (Figure 8).

### 6.1. Basic Inverse Perspective Mapping (IPM) Model

The objective is to map pixel points (u,v) to the world coordinate points (X,Y,Z). The notation $\left(\hat{\cdot }\right)$ indicates a vectored version of the variables. We first define a unit vector $\hat{X}$ to set the camera’s viewing direction. Being orthogonal to $\hat{X}$, we define another unit vector, $\hat{Y}$, that is orthogonal to the camera-viewing direction on the ground. The IPM is to find the relation between the world coordinate ($\hat{X},\hat{Y},\hat{Z}$) and image coordinate ($\hat{u},\hat{v}$) in order to map image pixels to the world coordinate points. Note that two types of coordinates on an image are set as ($\hat{u},\hat{v}$) and ($\hat{r}$, $\hat{c}$), depending on the unit. The relation between an image point in a pixel space ($\hat{u}$, $\hat{v}$) and the same point in a meter space ($\hat{r}$, $\hat{c}$) is defined as follows

(5) $$\begin{array}{ccc} u\left(c\right)=\frac{n+1}{2}+Kc & ⟷ & c\left(u\right)=\frac{1}{K}\left(u-\frac{n+1}{2}\right) \end{array}$$

(6) $$\begin{array}{ccc} v\left(r\right)=\frac{m+1}{2}-Kr & ⟷ & r\left(v\right)=\frac{1}{K}\left(\frac{m+1}{2}-v\right) \end{array}$$

with a scale factor between pixel and meter (px / m) K, the image width m, and the images height n. The location of the camera’s optical center (P) is [0,0,h] in the world coordinate system. The unit vector of the optical axis $\hat{o}$ is orthogonal to the image plane.

**Figure 9:**
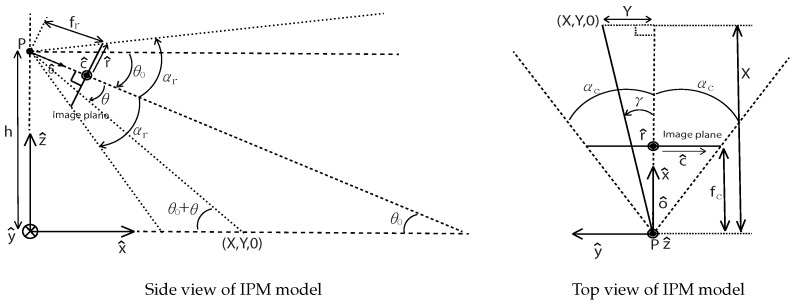
Side and top view of IPM model. In the illustration, $f_{r}$ and $f_{c}$ are the focal length of a camera; $\theta_{0}$ is the pitch angle; $\alpha_{r}$ and $\alpha_{c}$ are the half angle of the vertical and horizontal field of view (FOV) respectively. Reprinted, with permission, from Jeong et al. URAI 2016; ©2016 IEEE.

Using the top and side view in Figure 9 we can derive Equations (7) and (8) for a fixed camera case.

(7) $$\begin{array}{ccc} X(v) & = & h\frac{\tan\left(\theta_{0}\right)\left(1-2\frac{v-1}{m-1}\right)\tan\left(\alpha_{r}\right)-1}{\tan\left(\theta_{0}\right)+\left(1-2\frac{v-1}{m-1}\right)\tan\left(\alpha_{r}\right)} \end{array}$$

(8) $$\begin{array}{ccc} Y(u,v) & = & \left(1-2\frac{u-1}{n-1}\right)\tan\left(\alpha_{c}\right)X\left(v\right) \end{array}$$

The location of Y(u,v) in the world coordinate is dependent on (u,v) because Y(u,v) includes X(v).

### 6.2. Adaptive IPM Model

When images are obtained from a moving vehicle, it is difficult for them to be transformed to the accurate bird’s-eye view images because of the motion of the vehicle, especially its pitch direction. To resolve this problem, oscillatory pitch angle ($\theta_{p}$) of the camera is also added to original pitch angle ($\theta_{0}$) in this model. This oscillatory pitch angle can be obtained either from sensors or via visual odometry. In this work, we exploited IMU sensor for oscillatory pitch angle.

(9) $$\begin{array}{ccc} X(v,\theta_{p}) & = & h\frac{\tan\left(\theta_{0}+\theta_{p}\right)\left(1-2\frac{v-1}{m-1}\right)\tan\left(\alpha_{r}\right)-1}{\tan\left(\theta_{0}+\theta_{p}\right)+\left(1-2\frac{v-1}{m-1}\right)\tan\left(\alpha_{r}\right)} \end{array}$$

(10) $$\begin{array}{ccc} Y(u,v,\theta_{p}) & = & \left(1-2\frac{u-1}{n-1}\right)\tan\left(\alpha_{c}\right)X\left(v,\theta_{p}\right) \end{array}$$

Finally, the adaptive Inverse Perspective Mapping (IPM) modeling Equations (9) and (10) can be derived by adding $\theta_{p}$. $X(v,\theta_{p})$ is dependent on the pitch angle of the camera ($\theta_{p}$) and $Y(u,v,\theta_{p})$ is also dependent on it, which means that bird’s-eye view images are properly compensated against the pitch angle.

### 6.3. Consistent Lane Map Processing

As described in Section 5, the algorithm drops nodes based upon the wall extraction events. Depending on the circumstances, structure occurrence may be sparse and thus produce a large gap between nodes. When the distance between two consecutive nodes are usually a couple of meters, inter-node inconsistency for measurements becomes substantial. This is because the SLAM algorithm only corrects navigation error for the nodes. Since only nodes are optimized through SLAM update, uncorrected measurements needs post-processing for self-consistent maps. This discrepancy statistics is shown in Table 3 that describes pose error prior to compensation. The error is computed as the distance and angle between the node position that is generated by the proposed method and is the expected pose by using the vehicle encoder. The cause of this discrepancy is encoder error from the wheel slip and modeling error, and this large discrepancy clearly indicates the need for the compensation. Smooth vehicle poses corrected with SLAM data are obtained after this compensation process.

**Figure 10:**
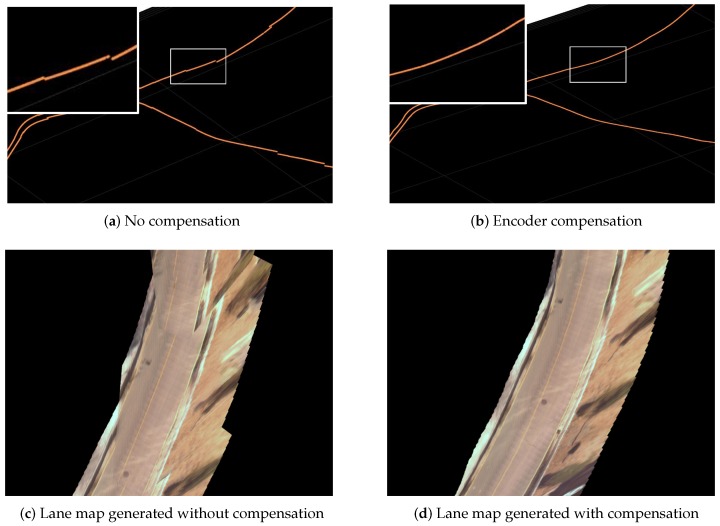
Result of self-consistent lane map process. Left (**a**,**c**) and right (**b**,**d**) images show the result of lane map.

The effect of compensation is presented in Figure 10. In our algorithm, valid SLAM nodes are sparse, and thus are not compact enough to create a lane map via IPM. Interpolation is required for a dense map by updating the navigation correction between the nodes using vehicle encoder data. Figure 10a shows interpolated positions between nodes without any compensation. There are lots of errors between expected positions using encoder data and nodes because all nodes are corrected when wall extraction events occur. To overcome this problem, we apply a compensation rule, as described below.

(11) $$\begin{array}{ccc} E_{k} & = & \frac{p_{e}-p_{n}}{n}k \end{array}$$

**Figure 11:**
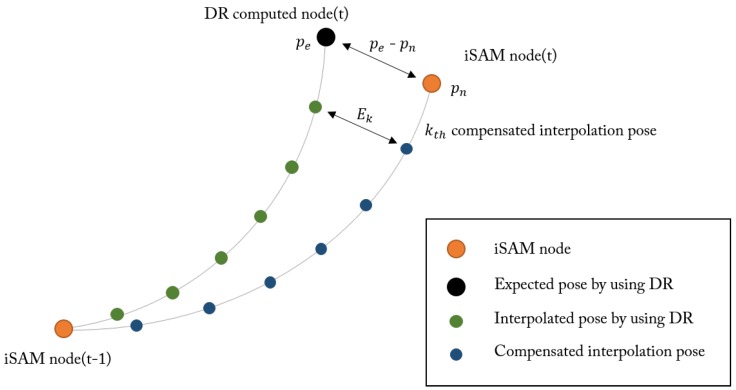
Compensation process of interpolated pose between DR computed node and iSAM node.

As shown in Figure 11, we compute the compensation value $E_{k}$ per section between nodes. For a section with two nodes on each end, we compared the Dead-Reckoned (DR) position $p_{e}$ and the SLAM-corrected node position $p_{n}$. When there are *n* number of images in the section we apply compensation value $E_{k}$ for the position associated with $k^{\mathrm{th}}$ image. The result of this interpolation is shown in Figure 10c,d. Note that the compensated projection demonstrates a more smooth and consistent lane map.

## 7. Experiments and Results

To validate the proposed method in the real world, we conduct an experiment covering a campus area with 9.32 km path length. The dataset is the route around a campus environment including four loop-closures by passing the center building repeatedly. The campus area is usually composed of low-rise buildings and wide roads that offer sufficient but sporadic GPS signals. As can be seen in Table 4, we have a 41.4% of GPS signal reception.

**Figure 12:**
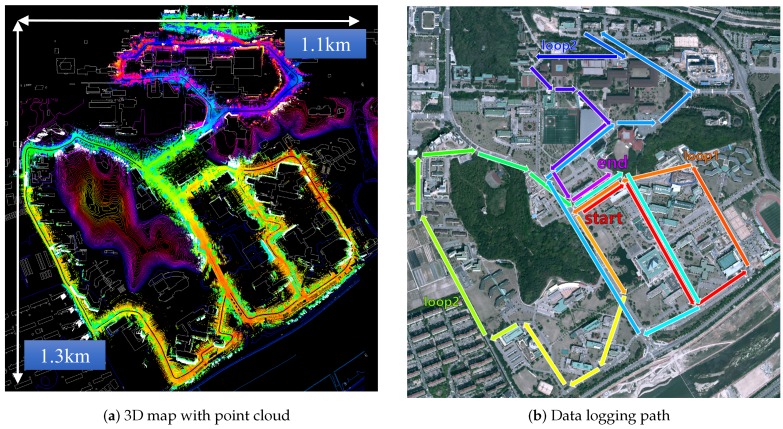
3D mapping result and data logging path. (**a**) 3D map result represented by pseudo-colored point cloud in digital map background. Nodes are color-coded by height varying from red (low) to purple (high). (**b**) Illustration of data logging path performed in the experiments. Connection of the trajectory represented by color shift from red (start) to purple (end). There are three circular paths covering the eastern, the western, and the northern part of the campus.

The overview of the entire campus area is as in Figure 12 depicted in the top-down and perspective view with a scale marked. In total 9.32 km of path is covered in 32 min. Overall the campus shows an altitude difference of 29.5 m between the northern and southern part. The final SLAM graph consists of 1165 nodes, and the average distance between the nodes is 8.06 m.

### 7.1. SLAM Results

**Figure 13:**
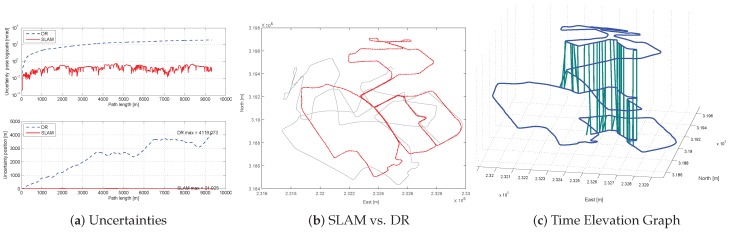
Proposed digital map-based SLAM results. (**a**) Uncertainty versus path length . Whereas the uncertainty of DR navigation (blue, dot) shows unbounded growth, the uncertainty of SLAM (red solid) is bounded from wall-to-wall loop closures. The graph at the top of the pose uncertainty with log-scale of y axis (unit: m·rad. The following [53,54,55], we use m·rad to show pose uncertainty), the bottom graph shows the position uncertainty (unit: m). (**b**) Top-down view of the SLAM estimate (red line) versus dead-reckoning trajectory (gray line). (**c**) The xy component of the SLAM trajectory estimate is plotted versus time, where the vertical axis represents mission time. Green lines show wall-to-wall matching for loop closure.

We first evaluate the SLAM results. Compared to Dead-Reckoned (DR), SLAM provides a more consistent map as shown in Figure 13b. The position is a value converted to a standard reference point (38${}^{\circ }$00’00”N/129${}^{\circ }$00’00”E), which follows the method of transverse Mercator coordinates. The algorithm was capable of making four times of loop closure successfully (Figure 13c) using a wall-to-wall loop-closing measurements. The node uncertainty propagation plot shows the SLAM effectively bounds the uncertainty (Figure 13a). We plot both the entire covariance determinant ($\sqrt[{6}]{\Sigma }$ in m·rad) and the positional covariance determinant ($\sqrt[{6}]{\Sigma_{xyz}}$ in m). DR shows approximately 10% of the positional uncertainty, while SLAM results are mutually bounded by wall-to-wall loop closure, GPS and digital map correction algorithm. In Table 4, the computation time with respect to the total mission time is summarized. The entire method runs in real time using only 40% of the total mission time. This is due to the fact that the low-rise building allowed more GPS signal reception , because the number of the GPS and the wall node was the critical factor in terms of process time.

**Figure 14:**
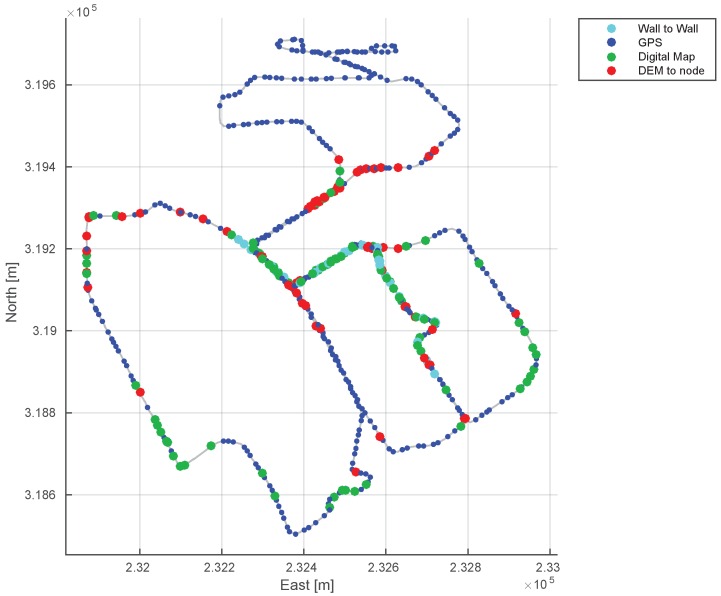
Sensor availability graph. The nodes are colored by the sensor type associated with the node. Blue dots are the GPS nodes and are in a smaller size for clear visualization for digital map nodes (green and red). Cyan nodes are loop-closure nodes via LiDAR comparison.

Figure 14 visualizes the nodes by the sensor types for the entire trajectory. As the environment is with low level buildings, GPS signals (blue) are available for many nodes (41.4%). However, the signal is sporadic and vulnerable to the surrounding structures. When the GPS signal becomes unreliable we use complementarity available structural information and correct against the digital map (green). DEM is also effective when altitude change is substantial within a region.

### 7.2. Qualitative Urban Mapping Results

For further validation of the proposed method, we back-project 3D point clouds and images using the accurate localization and mapping results. By doing so, we can generate a 3D urban map with lanes potentially for autonomous car driving. By confirming the consistency in the back-projected dataset, we validate the accuracy of the proposed method and application to urban map generation.

#### 7.2.1. 3D Point Cloud Map

**Figure 15:**
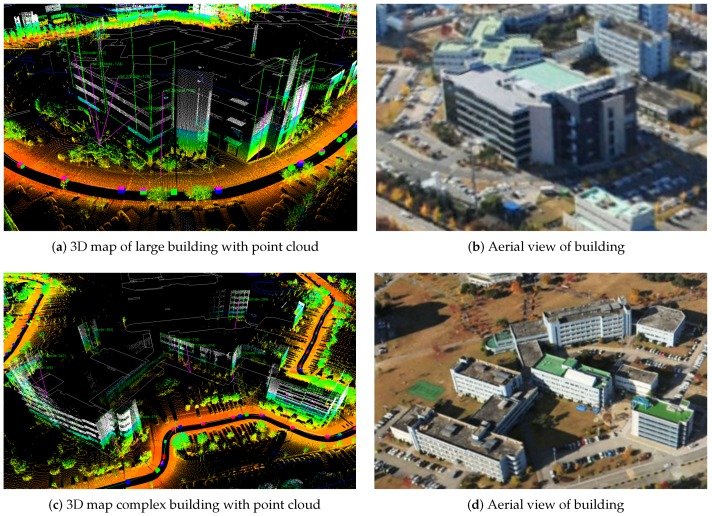
3D mapping result for sample buildings. Accumulated and refined point cloud using SLAM trajectory is given. (**a**) and (**c**) are the 3D mapping of two samples. Points are colored by the height, and green squares indicate the classified building walls. (**b**) and (**d**) are the aerial view of each sample building.

Collected LiDAR points are locally accurate with respect to the vehicle pose under exact extrinsic calibration. However, the entire map consistency may not be guaranteed if the estimated trajectory is with errors. Using the SLAM refined trajectory, we use point cloud information in order to create a 3D map of the urban environment. Figure 15 presents a qualitative result on two sample buildings created from the back-projection. As can be seen in the sample buildings of Figure 15, the proposed method is capable of detecting even highly complex walls The accuracy of the 3D modeling is significantly affected by the trajectory estimation accuracy. The SLAM based method enables accurate estimation even under a complex motion with many turns (Figure 15c).

#### 7.2.2. Lane Map via IPM

**Figure 16:**
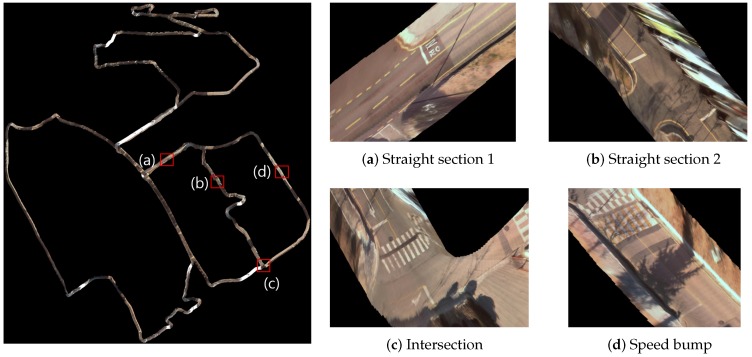
Generated lane map. (**a**) and (**b**) is a narrow straight section without and with parked car respectively. (**c**) is intersection and (**d**) includes speed bump and crosswalk.

We also generate a lane map for urban mapping. Using the Simultaneous Localization and Mapping (SLAM) results and the previously introduced adaptive Inverse Perspective Mapping (IPM) model, we produce precise bird’s eye view images compensating for vehicle motion. To generate dense and precise lane map, additional vehicle positions are required because the average distance between nodes of SLAM result is 8.06 m. Additional vehicle position and heading angle are interpolated by using vehicle encoder data from nodes that generated by SLAM. Figure 16 shows the entire campus lane map with four representative samples (shown in a zoomed view). Note the consistency of the generated map by the IPM images back-projection.

### 7.3. Accuracy Analysis

**Figure 17:**
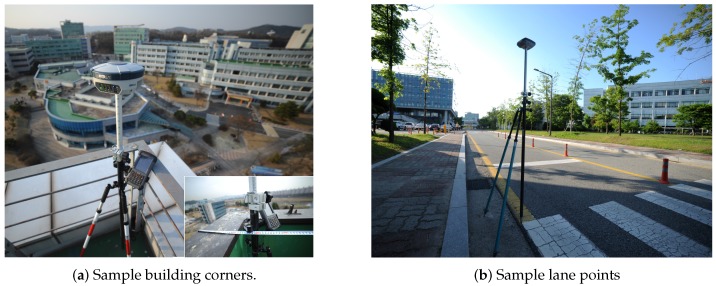
Accuracy analysis on sample points using VRS-GPS. (**a**) Four corners of the building rooftop are measured with building walls compensation. (**b**) Sample points on road marks are measured.

In the previous section, we have demonstrated consistency and qualitative evaluation. We also performed quantitative evaluation on the mapping results. Using RTK-GPS with 10 mm-accuracy, we analyzed the accuracy of the points from both sample buildings and lane maps. We use GRX2 by Topcon [56] in the RTK mode to measure ground truth point sampling. As shown in Figure 17, we measure sample points from both buildings and lanes.

**Figure 18:**
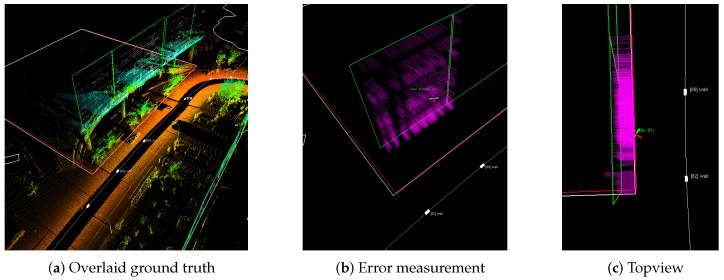
(**a**) Red square drawn from four sample ground truth corners. (**b**) By measuring perpendicular distance (purple line) from 3D point cloud to ground truth (red line) error is measured. (**c**) shows the topview for a clear illustration.

We analyze the effect of each measurement model for the entire map generation. Our evaluation process is described in Figure 18. Four building corners measured by RTK-GPS are connected to complete the boundary of a building (red square in Figure 18a. By measuring a perpendicular distance from LiDAR-extracted wall to the ground truth, we compute the Root Mean Square Error (RMSE) mapping error for 3D structures. The error analysis is summarized in Table 5. GPS-based method shows about a meter accuracy due to the sensor accuracy and unreliable availability. Compared to GPS-based mapping, the digital map-based SLAM shows substantial improvement over the conventional approach.

**Figure 19:**
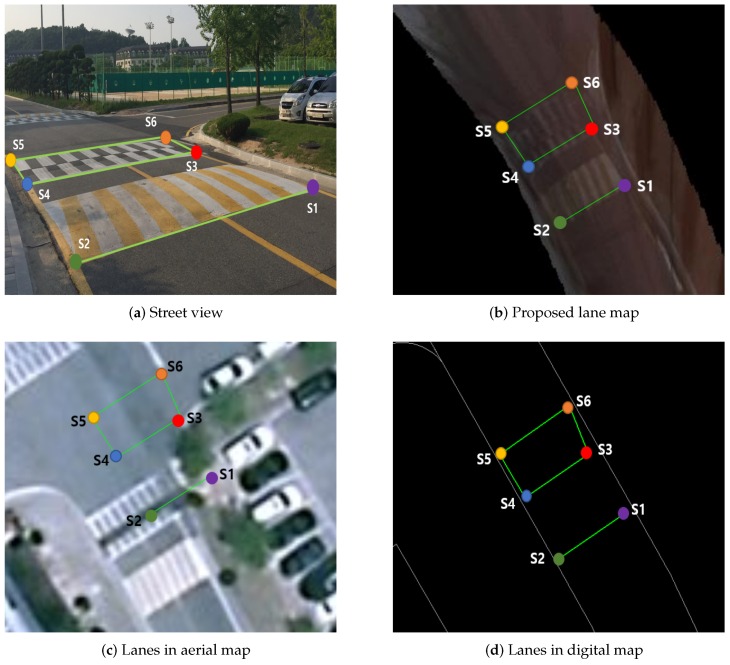
Six sample points having accurate global position are measured by RTK-GPS, and they are plotted on (**a**) street view, (**b**) lane map, (**c**) aerial map and (**d**) digital map. (**c**) Substantial position error with ground truth in aerial map, (**d**) Small position error with ground truth occurs, but lacking in detail such as road mark and cross walk. (**b**) Proposed method has position accuracy with global ground truth and detail information.

Similarly, we examine accuracy on a lane map from a set of sample points on road markings. In total, 16 points from five different sets are measured. Among them, Figure 19 presents validation process for a sample set with six sample points. Six RTK-GPS positions are obtained and used as ground truth (Figure 19a). In this analysis, we compare the proposed lane map with aerial image and digital map. Overlaying ground truth on aerial images (Figure 19c) reveals substantial discrepancy due to distortion without compensation from other sensors. In digital maps, road boundaries are accurate but road marking and lanes are missing. Compared to digital and aerial maps, the proposed method (Figure 19b) demonstrates substantial accuracy improvement.

Table 6 lists accuracy analysis for the proposed method for all five test sets. As in the 3D map analysis, we compare the RTK-GPS measured ground truth with aerial image and proposed method. Unlike the 3D structural map error, the SLAM error is not the only error source for this road marking error. When manually selecting a pixel from distorted and stitched road images, other factors related to image processing influences (e.g., image resolution, image quality, image distortion, manual pixel selection accuracy, and interpolated motion error). Lane parameterization and online conversion will surely improve overall lane results from fine refinement. In this work, however, we mainly aim to validate the SLAM result accuracy by examining error from structural and lane mapping results.

## 8. Conclusions

This paper proposes a seamless way to incorporate publically available digital maps and sensors mounted on a mobile platform. For accurate urban mapping, we focus on 3D structural and lane mapping. The proposed method tackles the accurate map building problem by integrating global measurements with local sensor measurements via Simultaneous Localization and Mapping (SLAM). The results validate the accuracy of the created map. As the ground truth is not available we have conducted accuracy analysis by measuring a set of sample points using RTK-GPS. The proposed method has proven itself capable of balancing unreliable GPS and digital map information to create an accurate map.

## Figures and Tables

**Table 1:** Specifications of Urban Mapping System (UMS).

Item	Specification
Dimensions	1.67 m × 1.36 m × 0.31 m (L × W × H )
Dry weight	35.8 kg
LiDAR	SICK LMS291,200 (35 Hz)
Imaging sensor	Point Grey Flea3, 1380 × 1024 pixel, 12-bit CCD (30 Hz)
GPS	HUACE B20 (1 Hz)
IMU sensor	Xsens MTi (100 Hz)
Altimeter	WITHROBOT myPressure (1 Hz)
Wheel encoder	Autonics E68S, rotary encoder type (100 Hz)
Processor	Intel(R) Core(TM) i7-3790 CPU@3.4 GHz
Battery	Delkor 80 Ah, 12 V , lead–acid type

**Table 2:** Description on polygon record contents. The fields for a polygon type are box, numParts, numPoints, parts, and points. Box means the bounding box for the polygon stored in the order Xmin, Ymin, Xmax, Ymax. The number of closed curves in the polygon is described by NumParts. NumPoints is the total number of points for all closed curves. parts means an array of length numParts. For each closed curve, the index of its first point stored in the points array. Points are array of length numPoints. The points for each closed curve in the polygon are stored end to end.

Position	Field	Value	Type	Number	Byte Order
Byte 0	Shape Type	5	Integer	1	Little
Byte 4	Box	Box	Double	4	Little
Byte 36	NumParts	NumParts	Integer	1	Little
Byte 40	NumPoints	NumPoints	Integer	1	Little
Byte 44	Parts	Parts	Integer	NumParts	Little
Byte X†	Points	Points	Point	NumPoints	Little
†X = 44 + 4 × NumParts					

**Table 3:** Discrepancy prior to the compensation (per unit meter).

	Min	Max	Average
x (m)	$7.98\times 10^{-4}$	$9.37\times 10^{-2}$	$1.99\times 10^{-2}$
y (m)	$3.64\times 10^{-4}$	$1.49\times 10^{0}$	$4.13\times 10^{-2}$
yaw (${}^{\circ }$)	$2.08\times 10^{-3}$	$2.47\times 10^{1}$	$1.58\times 10^{0}$

**Table 4:** Summary of Digital-Based SLAM results.

Path Length	Logging Time	Computation Time	GPS Node	Wall Node	Digital Map Node	Total Nodes	No. of Point
9322.35m	1952.06s	792.04s (40.57%)	482 (41.4%)	249 (20.09%)	136 (11.67%)	1165	23,017,120

**Table 5:** Positional error measurement between the 3D building point cloud and RTK-GPS measured building corners. The map error is computed from average of RMSE between the ground truth wall generated from RTK measured sample points and LiDAR 3D points in the mapped wall. Note that this error is over 9.32 km of travel distance.

Sample	No. of 3D Map Points	Average RMSE	
		GPS Only [m]	Digital Map-Based [m]
Set 1	3352	0.437	0.190
Set 2	1967	1.010	0.193
Set 3	227	2.070	0.347
Set 4	3314	1.407	0.136

**Table 6:** Error analysis for the proposed method on road marking. Each dataset has two to six sample points respectively. For set 2, no road marking in the aerial map and excluded in the comparison. The proposed method’s error is also written as a ratio to the aerial image for clear comparison.

Sample	No. of Lane Points	Average RMSE	
		Aerial Image [m]	Proposed Method [m]
set 1	2	7.180	1.000 (13.927%)
set 2	2	-	1.055 ( - %)
set 3	2	8.948	1.699 (18.989%)
set 4	6	8.044	0.622 (7.727%)
set 5	2	11.261	0.610 (5.418%)
